# Supplementary material for: Identification of Medicinal Compounds of Fagopyri Dibotryis Rhizome from Different Origins and Its Varieties Using UPLC-MS/MS-Based Metabolomics
Source: Metabolites. 2022 Aug 25;12(9):790. doi: 10.3390/metabo12090790 (PMC9503457; doi:10.3390/metabo12090790)
Supplement: Supplementary file 1 [file metabolites-12-00790-s001.zip › Supplementary Figures.pdf]

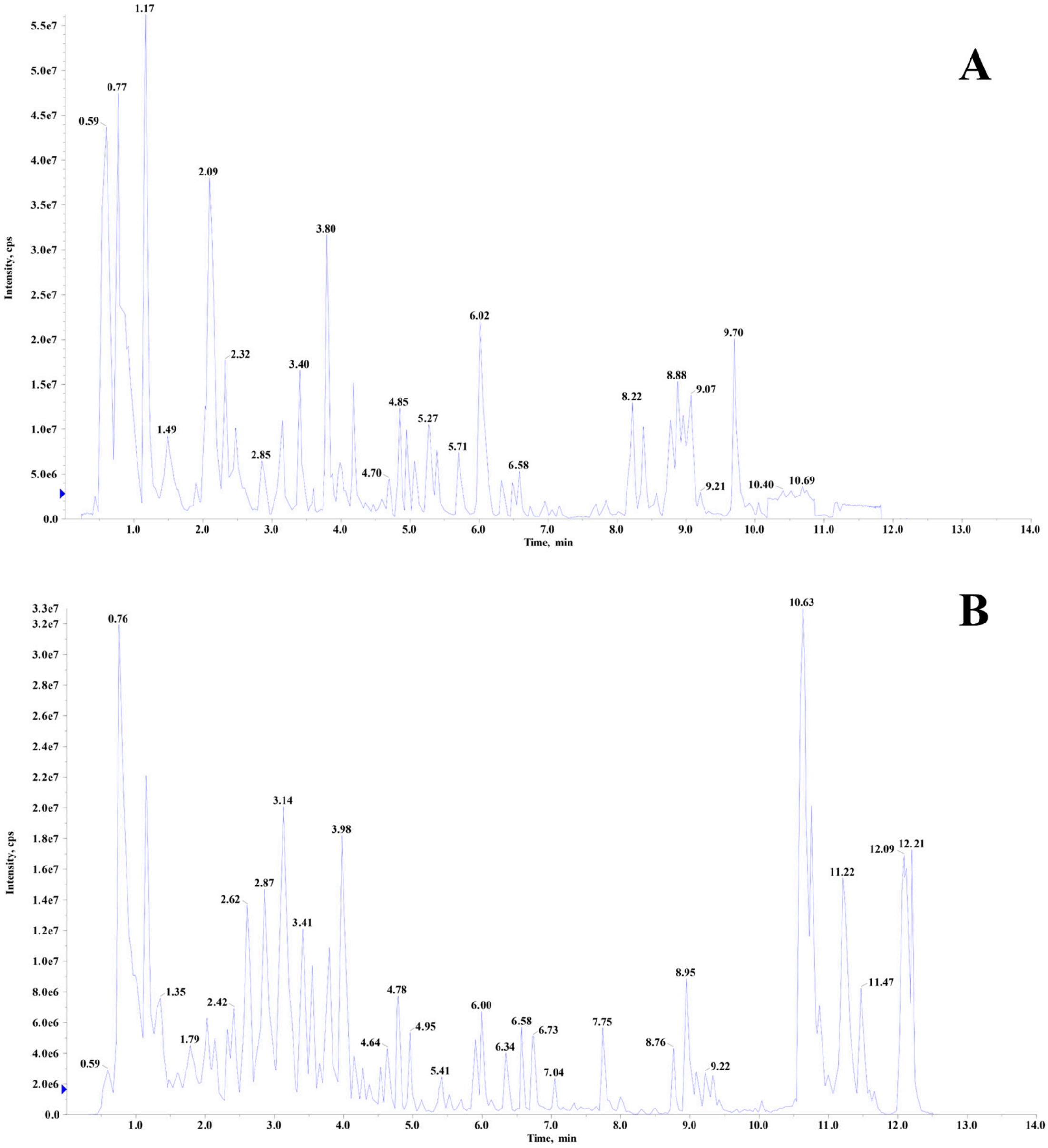

Figure S1. The stacking diagram of total ions current (TIC) maps from quality control samples (QC) mass spectrometry. **(A)** TIC of positive ion multiple reaction monitoring (MRM). **(B)** TIC of negative ion MRM

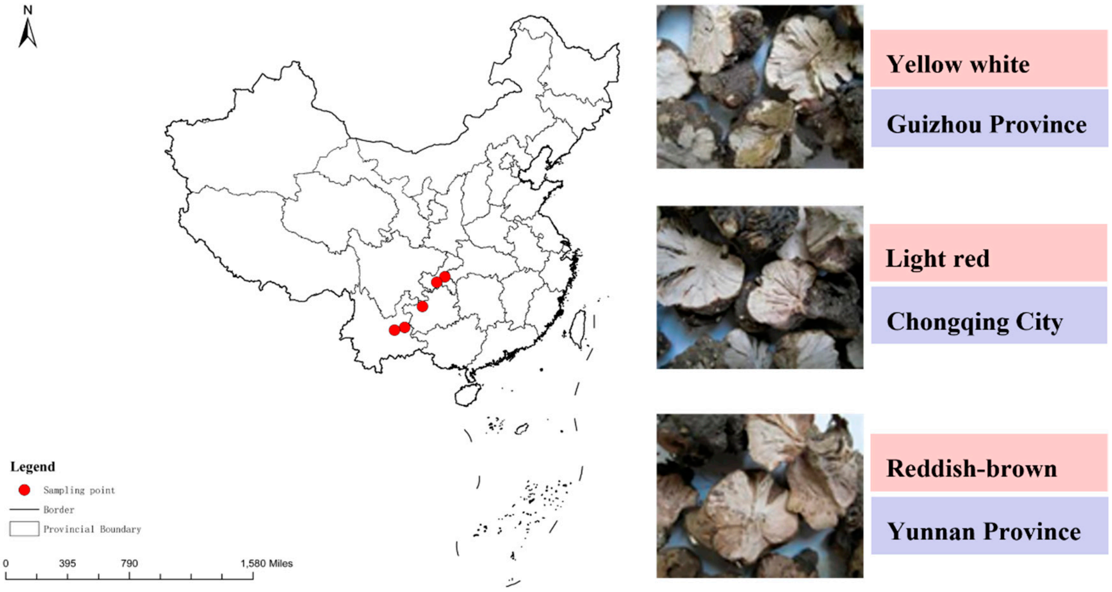

Figure S2. Cross-section of FDR from different producing areas. **(A)** YK (Reddish-brown); **(B)** CS (Light red); （**C**）GB (Yellow white).
