# Supplementary material for: Identification of Medicinal Compounds of Fagopyri Dibotryis Rhizome from Different Origins and Its Varieties Using UPLC-MS/MS-Based Metabolomics
Source: Metabolites. 2022 Aug 25;12(9):790. doi: 10.3390/metabo12090790 (PMC9503457; doi:10.3390/metabo12090790)
Supplement: Supplementary file 1 [file metabolites-12-00790-s001.zip › Supplementary Materials and Methods.pdf]

2. Materials and Methods (Supplementary material)

2.3.1. Settings of the Ultra-Performance Liquid Chromatography assays.

We analyzed the FDRs extract samples using an UPLC-ESI-MS/MS system (UPLC, SHI MADZU NextEra X2, MS, 4500 Q TRAP; Applied Biosystems). The conditions for the liquid phase analyses were set as the following: the chromatographic column of UPLC equipped with a C18 column (Agilent SB-C18, 2.1 mm × 100 mm, 1.8 μm). Pure water containing 0.1% formic acid was named the solvent A; acetonitrile containing 0.1% formic acid was named the solvent B; the solvents A and B were used as the mobile phase with a gradient setting as the following: the starting condition was 95% A, 5% B. Then, within the next 9.00 min, the proportion of the solution B gradually increased linearly to 95%, then, this composition was kept for 1.00 min; then, during 10.00 to 11.10 min, the proportion of solution B was set to decrease to 5%. The settings would be equilibrated with 5% solution B till 14 min. Column temperature was set as 40 ℃. Flow velocity was set as 0.35 mL/min. Injection volume was 4 μL. Effluent was connected to an ESI-triple quadrupole-linear ion trap (QTRAP)-MS alternatively [18].

2.3.2. ESI-Q Trap-MS/MS

We detected metabolites based on an AB4500 Q TRAP UPLC / MS / MS system, which was equipped with triple quadrupole (QQQ) scans and linear ion trap (LIT). A triple quadrupole-linear ion trap mass spectrometer (Q TRAP) was used as survey scans. This system was equipped with an ESI turbo ion-spray interface, which could be operated via the Analyst 1.6.3 software (AB Sciex) in both the positive and the negative ion modes. The chromatogram for each elution period is shown in Figure S1. The ESI source operation parameter was an ion source (turbo spray, 550 °C), and had two ion modes (5500 V (positive ion mode) / - 4500 V (negative ion mode)). Ion source gas I (GSI), gas II (GSII) and curtain gas (CUR) were set to 50, 60 and 25.0 psi, respectively, and the collision-induced dissociation parameters was set to high. In the QQQ and LIT modes, 10, and 100 μmol/L polypropylene glycol solutions was used for the instrument tuning and mass calibration, respectively. We used the multiple reaction monitoring (MRM) experiments with collision gas (nitrogen) set to medium for QQQ scans. We accomplished the de-clustering potential (DP) and calibration (CE) of each MRM transition through further DP and CE optimization. According to the metabolites eluted in each period, a specific set of MRM transitions was monitored.

2.4.1. Qualitative and Quantitative Metabolite Analyses.

The qualitative analysis of metabolites was performed based a self-compiled database, MWDB (Met Ware Biological Science and Technology Co., Ltd, Wuhan, China) [18]. According to the information of secondary spectrum, the repeat signals of K^+, Na^+, $NH4^+$, and other substances with large molecular weights were eliminated. The quantitative analysis of metabolites was performed in the multiple reaction monitoring (MRM) mode of QQQ. In the MRM mode, the characteristic ions of each metabolite were screened through the QQQ-MS to obtain the signal strengths. After obtaining the metabolite profile data of different samples via the mass spectrometry analyses, integration of the chromatographic peaks of all substances were conducted by MultiQuant software 3.0.3, and the integration and correction of mass spectrum peaks detected by each metabolite in different samples were also carried out [19]. Finally, all the chromatographic peak area integral data were derived, and the relative content of the corresponding metabolites were calculated as the peak area integrals [20].
